# Linguistic stereotyping in natural language: How and when we generalize in communication about people

**DOI:** 10.1080/15456870.2025.2525799

**Published:** 2025-07-17

**Authors:** Camiel J. Beukeboom

**Affiliations:** Department of Communication Science, Vrije Universiteit Amsterdam, Amsterdam, the Netherlands

## Abstract

Language plays a key role in shaping and maintaining social-category stereotypes. While past research often examined isolated linguistic features in experimenter-generated sentences, in largely independent fields, this article takes an integrative approach by focusing on how stereotypes are communicated in natural, freely generated language. As social-category stereotypes are generalized impressions, a central mechanism in linguistic stereotyping is generalization, expressed in language that generalizes across individuals, situations, and time. I first examine how generalization takes shape in communication about others, and how this results in stereotype formation in recipients. Next, I explore when people generalize in their communication, rather than specify and use more nuanced descriptions about individual, situated behavior. Together, this reveals how everyday language perpetuates stereotypes in society. In conclusion, I discuss potential future research directions.

An important question in the field of intergroup communication is how social-category stereotypes are formed and maintained within (sub)cultures (Giles et al., prolog this volume). Language use plays a crucial role. In everyday communication, we frequently introduce social categories (Kurylo & Hu, 2024; Whitehead et al., 2024). We may discuss the characteristics of genZ, the generic differences between men and women, talk about streams of immigrants, or people labeled as woke. Using language we not only create categories but also form generalized impressions about them. Through language, we shape the way we perceive the social world (Boroditsky, 2010; Enfield, 2022).

Existing research on stereotype communication and linguistic bias has shown the various ways in which language reflects and maintains social-category stereotypes. When communicating about other people and their behavior, the speaker’s linguistic choices (in both labeling and behavior descriptions) tend to reflect activated social-category stereotypes. Moreover, such linguistic expressions feed social-category perceptions in message recipients (Beukeboom & Burgers, 2019). Most research, however, has experimentally examined linguistic features in isolation (e.g., labels, abstraction, negations), either testing stereotype expression in language by having participants rate or choose experimenter-generated sentences or by testing recipient inferences from manipulated linguistic variations. Research on stereotype communication in freely generated natural language^1^^1^The term ‘natural language’ is used to refer to human-produced language in which people are free to choose their own words and generate their own formulations. Such ‘freely generated, natural language’ exists in everyday conversation, in social media or news content, but also in experiments in which participants generate their own descriptions (as opposed to rating experimenter-generated sentences). remains scarce (Beenen et al., under review). To further advance the gained insight about the role of linguistic features in stereotype communication, a more integrative approach is now needed to understand these processes in real-life discourse (Beukeboom & Burgers, 2019).

In natural language, people use creative and more complex linguistic constructions than what is typically seen in isolated experimentally controlled single sentences. Natural language texts are longer; descriptions may develop from discussing situational behaviors of one or a few categorized individuals to generalizations about a generic category, and various known linguistic biases in behavior descriptions may interact or occur simultaneously. Moreover, various contextual factors (e.g., genre, medium, interpersonal, and intergroup relationship toward the discussed target and conversation partner) may determine whether stereotypes are expressed and/or result in stereotype formation in recipients.

In this article, I aim to integrate insights from communication science, linguistics, social- and developmental psychology, to gain a better understanding of linguistic stereotyping in everyday natural language use. I approach this by proposing linguistic generalization as a foundational mechanism in the communication stereotypes, which are, by definition, generalized impressions. This integrates various known linguistic biases within a unified explanatory framework. I first examine how generalization takes shape in communication about others, and how this results in stereotype formation in recipients. Next, I explore when people generalize in their communication, rather than specify and use more nuanced descriptions about individual, situated behavior.

An in-depth understanding of linguistic stereotyping in real-life contexts can uncover underlying patterns of social categorization and reveal the stereotypical expectations we hold. Awareness of these patterns can facilitate prevention of unwanted effects, inform interventions, as well as research in applied contexts. The latter becomes even more appealing considering the rapid development of computerized natural language processing (NLP) techniques (Boyd & Markowitz, 2025). To effectively harness the growing toolkit of NLP methods, it is essential that these tools are grounded in a robust transdisciplinary theoretical framework (Boyd & Markowitz, 2025) – a foundation to which this article seeks to contribute. Ultimately, this will offer a more comprehensive understanding of how language contributes to the perpetuation of stereotypes, prejudice and discrimination in society.

## Social categories, stereotypes, and stereotyping

Decades of research has demonstrated the fundamental and ubiquitous effects of social identity, categorization, and stereotypes in social perception and interaction (Allport, 1954; Tajfel & Turner, 1986; Turner et al., 1987; Zhang & Harwood, this volume). Social categorization and reliance on stereotypes is generally seen as functional. Identifying individuals as belonging to a social category allows perceivers to draw on beliefs and expectancies about similar individuals from that category (Mackie et al., 1996). The automatically activated category stereotype^2^^2^The term stereotype refers to the cognitive representation people hold about a social category, consisting of beliefs and expectancies about probable behaviors, features, and traits (Dovidio et al., 2010). This cognitive component can be distinguished from an affective or evaluative response toward a social category (Amodio & Devine, 2006). The term ‘prejudice’ usually refers to negative affective evaluations of a social category and its members. These cognitive and affective associations may (in concert or independently) induce discriminatory behaviors (Amodio & Devine, 2006). provides us with associated properties that go ‘beyond the information given’ (Allport, 1954), which we can use to quickly and efficiently make sense of a complex social environment. Yet, the simplification it brings has serious downsides. When we have categorized, we seem to relax our cognitive efforts and attention (Enfield, 2022), and relying on generic stereotypic knowledge leads to superficial judgments.

This is first because categorization into a single category reduces an individual or a group of individuals to just one dimension. It (at least temporarily) ignores all the potential other intersectional categorizations that may apply to these persons. Second, when relying on a category and its associated stereotype, perceivers both exaggerate similarities between individuals within categories (i.e., they are seen as all alike) and differences between categories (Allport, 1954; Brewer & Harasty, 1996). Third, while judging a categorized person based on stereotypic expectancies provides information gain (i.e., stored category cognition is applied to this individual), it also involves information loss, because the individuality and situational constraints of category members are ignored (Fiske, 1998; Mackie et al., 1996; Macrae & Bodenhausen, 2000). In other words, when we think about and judge people on generic expectancies, we fail to see that their social identity consists of multiple potential alternative categorizations and that behaviors and characteristics can vary in time, place, situation, and context.

## The importance of language: stereotyping in discourse

To understand how social-category stereotypes evolve, are maintained, and how and when they are used, it is crucial to focus on language use (Collins & Clément, 2012; Mackie et al., 1996; Whitehead et al., 2024). Social categories and stereotypes are, in essence, products of collections of individuals that are formed and maintained by – and consensually shared among – people within (sub)cultures (Haslam et al., 1997; Holtgraves & Kashima, 2007; Kashima et al., 2010). By communicating about others and ourselves – which we do continuously – we collaboratively make sense of our social world and our own role in it. Language allows humans to communicate about social actors and events they did not observe themselves and to share both factual observations and interpretive impressions about them. Moreover, language allows us to go beyond one-time observed behavioral events and to generalize across individuals and specific situations. A generic belief about a broad social category, like ‘Girls are sweet,’ cannot be observed directly, nor can this information be illustrated for someone else without the use of language (Gelman et al., 2004).

Using linguistic references, we designate and label individuals and smaller or larger groups. This way we carve up reality and communicate which categories we deem relevant. Moreover, when we communicate about what we perceive in and expect from the categories we created, we collaboratively form generalized stereotypic impressions. Finally, we use language when we apply these stereotypes to new individuals behaving in new situations (i.e., stereotyping). We discuss individuals or subgroups that conform to these generalized expectancies, and we try to make sense of those who deviate from it (Abrams & Lalot, this volume). Even though other, non-linguistic factors play a role in the formation of categories and stereotypes (e.g., appearance or dress styles, interactions with category members, visual depictions of categories in the media, or perceived segregation within a society), even these factors likely co-occur with linguistic categorization in communication. After all, for the formation of shared social-category perceptions, social verification is needed, which is generally achieved through linguistic communication (Echterhoff et al., 2005). In other words, it is mainly through language that we collaboratively form and communicate generalized stereotypic impressions of categories of people.

This has an important function, because this way we create a shared reality with others in our (sub)culture, which is needed for social coordination, joint decisions, and actions (Enfield, 2022). By sharing and discussing information and impressions about others, we monitor and verify the veracity of our social-category cognition and create a common ground in how we perceive our social reality (Kashima et al., 2007; Klein et al., 2008).

## An integrative approach: the social category and stereotype communication (SCSC) framework

The social category and stereotype communication (SCSC) framework (Beukeboom & Burgers, 2019) provided a first attempt toward an integrative view on how stereotypes are communicated in everyday freely generated, natural language. Based on an integrative literature review, the SCSC framework poses that biases in language use in communications about categorized individuals are linked to three fundamental variables in shared social-category cognition: perceived category entitativity, stereotype content, and essentialism.

Perceived category entitativity is defined as the extent in which a category is perceived as a meaningful, unified, and coherent group, as opposed to a loose set of individuals (Beukeboom & Burgers, 2019; Campbell, 1958). Particularly in language reception studies, it has been shown that linguistic references – and specifically strong labels like generic nouns (e.g., immigrants are …) – increase the perception that the category is a unified and coherent entity that is distinguishable from other categories within a social-category taxonomy (e.g., Carnaghi et al., 2008; Gelman & Heyman, 1999). Moreover, recent language production studies (Beukeboom et al., under review) demonstrate that the use of generic references (as opposed to a focus on individuals and subgroups) increases with higher perceived entitativity.

Stereotype content refers to the content of the cognitive representation people hold about a social category, consisting of beliefs and expectancies about probable behaviors, features, and traits (Dovidio et al., 2010; Zhang & Harwood, this volume). Research suggests that stereotype content and positive or negative prejudice are expressed in, and inferred from, the terms used (i.e., their semantic meaning and valence) to label social categories and to describe their behavior and characteristics (Croom, 2013; Nicolas et al., 2022).

Perceived category essentialism finally refers to the extent in which an associated set of characteristics and traits (i.e., stereotype content) is perceived to be immutable to its members, and stable across time and situations (Beukeboom, 2014; Beukeboom & Burgers, 2019). Research on various linguistic biases in communications about behaviors and characteristics revealed how subtle variations in language (e.g., negations rather than affirmations, and concrete rather that abstract predicates) communicate that a described behavior is a one-time exception to the rule (vs. typical and enduring) and thus induce lower levels of perceived essentialism (Beukeboom et al., 2010). Research on the linguistic expectancy bias (Maass, 1999; Wigboldus et al., 2000), for instance, demonstrates that stereotype inconsistent (versus consistent) information is described at a lower level of abstraction, which in turn results in lower perceived essentialism in message recipients.

In sum, the SCSC framework suggests that both perceived entitativity, stereotype content, and essentialism are reflected in, and inferred from language use. Stereotypes become stronger with a highly essentialized stereotype content, and this is most likely to occur with higher perceived category entitativity. It is thus highly relevant to understand which linguistic elements reflect, maintain, and strengthen these fundamental variables. The SCSC framework (Beukeboom & Burgers, 2019) distinguishes two linguistic bias types in (1) references/labels and (2) behavior descriptions, each of which is further specified by focusing on linguistic content and form. In natural language, these biases occur in combination, as I will illustrate in the next section.

## Generalization in language as a central mechanism

Social-category stereotypes are generalized impressions. That is, a strong stereotype consists of a set of essentialized features and characteristics associated with a social category, which is expected to apply to the whole category (i.e., many or all individual category members) and to be stable across situations. This means that the most direct expression of category stereotypes occurs in communication that generalizes across individuals and across situations and time. Generalized communication (as opposed to more nuanced, specific communication which conveys individual situated behavior) is thus fundamental to stereotype communication. I discuss it here, as a way to integrate the various aspects of biased language use under one umbrella.

In communication about categorized individuals, we distinguish two main dimensions in which generalization can occur: (1) in references to (a) target(s), and (2) in descriptions of behavior and characteristics. In referring to targets we can, on the specific end, focus on specified individual[s] or, on the generic end, focus on a generic social category which generalizes across individual category members. In describing behaviors and characteristics of (categorized) individuals we can, on the specific end, focus on concrete situational behavior, or, on the other generic end, focus on enduring characteristics and traits, which generalizes across situations and time (Beike & Sherman, 1994; Beukeboom & Burgers, 2019).

To understand how people generalize in language, it is informative to look at theory about abstraction. Abstraction has mostly been discussed as a cognitive phenomenon, focusing on how people learn, process, and cognitively represent information in categories (for an overview, see Burgoon et al., 2013). At the same time, language use goes hand in hand with these cognitive processes^3^. Some have even described language as a ‘categorization engine’ (Enfield, 2022), as it facilitates representing specific information in simplified broader categories.

Bolognesi et al. (2020) distinguish two dimensions of abstraction, which, they demonstrate, are to a certain extend orthogonal to each other. The first dimension is *categorical specificity*. It is defined as the gradable property that characterizes concepts on a scale from highly generic (e.g., concepts referring to broad groups or classes; e.g., animal) to highly specific (e.g., concepts referring to more specific groups or exemplars; e.g., Rottweiler). In other words, it describes how inclusive the category of reference is. This can be measured as the relative position of a concept within a taxonomy (Rosch et al., 1976), for instance, the WordNet taxonomy (Fellbaum, 1998).

When applied to stereotype communication, categorical specificity most easily links to references to targets. Referenced categories vary in how broad or narrow they are (Anderson, 1991; Rosch et al., 1976). Categories can range from superordinate broad-level categories (e.g., gender, age groups, ethnic groups), to more narrow subordinate levels of aggregation (e.g., specific professions, like surgeon). But also, within social categories, references can vary in specificity, by referring to a generic category as a broad entity (e.g., Germans are …), a subordinate subtype (e.g., female Germans are …), a subset (e.g., these Germans are …), or individual exemplars (e.g., This German is…).

The second abstraction dimension distinguished by Bolognesi et al. (2020) is *concreteness*. This is defined as the gradable property that distinguishes concepts on a scale from being characterized by lots of sensory-based information (i.e., highly concrete) to virtually none (i.e., highly abstract). In other words, it measures the degree to which a referent is tangible and can be perceived through sensory experiences (e.g., sight, touch, hearing). For example, ‘a furry dog’ is more concrete than ‘friendly animal,’ as it presents a more readily observable, detailed, and vivid image (Burgoon et al., 2013).

Applied to social information and stereotypes, this dimension also relates to references. A generic social-category reference (e.g., Girls are …) is very abstract, the referent cannot be experienced through our senses, it cannot be seen or touched. When we move to more specific referents, it also becomes increasingly concrete and tangible. Specified subgroups within a category (e.g., these girls), and individual exemplars (e.g., This girl Lucy) can more vividly be seen and touched.

The concreteness dimension can also be applied to descriptions of behaviors and characteristics of categorized individuals. A well-known approach within the social psychology field that has done exactly this, relates to language abstraction as defined by the Linguistic Category Model (LCM; Semin & Fiedler, 1988, 1991). This work argues that any behavior can be described in varying degrees of abstraction. The LCM describes four categories of predicate types with increasing abstraction. Descriptive action verbs are the most concrete and can be used to convey a description of a single, observable action with a physically invariant feature (e.g., ‘A hits B’). These descriptions retain a reference to the contextual and situated features of an event. The second category (interpretive action verbs and state action verbs) describes a general class of actions (e.g., ‘A hurts B’). It is more abstract in that this category of actions can be performed in multiple ways. The next category (state verbs) typically describes an unobservable mental state and not a specific event (e.g., ‘A hates B’). Finally, the most abstract predicate types in the LCM are adjectives (e.g., ‘A is aggressive’). These describe traits that generalize across events and objects and show no reference to context or to observable situated acts. Thus, while more abstract descriptions of behavior are more informative about the target’s enduring qualities (dispositional traits), it is less informative about specific situations, less verifiable, and more likely to induce disagreement (Semin & Fiedler, 1988, 1991).

Note that both the categorical specificity and the concreteness dimension (Bolognesi et al. (2020) can be applied to variations in descriptions of behaviors and characteristics. Behavioral events can also be categorized. When descriptions move from descriptions of observable actions (e.g., He hits B), to a category of actions (e.g., He fights B), to abstract traits (e.g., He is aggressive), the behavior is categorized in increasingly inclusive categories. The predicates become more and more inclusive in encompassing an increasing multitude of specific behaviors (i.e., decreasing specificity). The descriptions also become less perceptible, tangible (i.e., more abstract/decreasing concreteness) as the reference to observable actions in specific situations disappears.

For mental states (e.g., He hates B), the link with concreteness is also clear. Describing a behavioral situation with reference to mental states is more interpretive, and less perceptible than actions (i.e., it cannot be seen or touched), but it is more tangible than a dispositional trait). The link with the categorical specificity dimension is less clear as it does not relate to a more inclusive categorization than a description of an action.

In sum, when we generalize in communication about other people and their behavior (e.g., Girls are sweet), we place both individuals and behavior in categories. This way, we move away from communicating observable situated actions of specified individuals and instead generalize across individuals and across situations and time. Moreover, we convey an enduring and stable stereotypic impression of a social category depicted as a generic entity.

## The communication of stereotypes through generalization in natural language

How then do people generalize in everyday natural language, and when does it result in stereotype formation in recipients? In natural language, stories or conversations can entirely occur at a generic level; i.e., people may communicate generalizations about a category without any reference to specified persons or situations (e.g., the Japanese are really industrious). Communication becomes more nuanced and specific when discussing situational behaviors of subgroups and/or one or more categorized individuals (e.g., Mr. Sato routinely stayed late in the office). Or it can go back and forth. Descriptions of the situational behavior of an individual category member, for instance, can be invoked as evidence or illustration for inferences about stereotypic characteristics of a generic social category (e.g., Lucy helped her mother yesterday; girls are really helpful; Ruscher, 1998). Depending on the communicative context, such dynamics can become interactive. In dyadic conversation, a speaker’s utterances about specific behavior of individual category members can induce cognitive inferences and linguistic utterances at a generic level (and vice versa) in a conversation partner (Ruscher & Hammer, 2006).

To study how these dynamics work, we should look at both the use of references (i.e., to generic target categories, subgroups, or categorized individuals), as well as to variations in descriptions of their behavior and characteristics. Experimental research has mostly relied on carefully constructed sentences, focusing either on isolated variations in references/labels or in behavior descriptions (Beukeboom & Burgers, 2019). In contrast, in natural language people use a large variety of creative linguistic constructions to achieve their communicative goals. It is then important to distinguish between *what* is done/intended with a certain linguistic construction (i.e., the speech act, referring to actors or other entities, describing behaviors or characteristics) from *how* it is linguistically constructed (i.e., grammar, specific word choice, verb types, and adjectives). The linguistic context of an utterance is needed to grasp what is intended. When it comes to the communication of stereotypes, we are at first interested in *what* people do with their language (e.g., the extent of generalization across individuals and/or situations), and only secondary in the linguistic means to achieve this (the how).

How do references work in natural language? A linguistic reference is an expression that is used to refer to (or designate/identify) an entity in the (real or imagined) world and can consist of one or multiple words. In linguistics terms, references are commonly expressed in noun phrases, which are units (or constituents) that fulfil a syntactical function in a sentence (e.g., grammatical functions like subject, object, and/or semantic roles like agent, patient).

References can be pronouns (e.g., they, he), substantive nouns (e.g., girls) or proper nouns (e.g., Lucy), but also longer noun phrases including articles, adjectives (e.g., a nice girl) or verbs and/or prepositional phrases (e.g., the girls walking in our street) which function to modify the head noun. Illustrating their fundamental importance, research shows that already at a preschool age, children are sensitive to subtle cues that indicate which entity is referred to, being a generic category, subgroup, or exemplar (e.g., cats have tails, the cats have tails, the cat has a tail; Cimpian & Markman, 2008).

In stereotype communication, multiple variables related to references are important. The level of specificity of the referent is important (i.e., generic category, subgroups, or individuals). In longer texts or conversations, most references will be pronouns. A speaker likely first introduces one (or more) labeled entity/entities (e.g., referring to a group or individual, using (modified) nouns or descriptive references (e.g., the nice girl(s) walking in our street) and continues with pronouns (e.g., she, they) that refer to the previously introduced labeled entity. At a text level, the number of co-reference chains can reflect how many different referents are introduced. A text that focuses on merely discussing a generic category (Girls are … , They …) has just one co-reference chain referring to one entity. A more nuanced text would introduce multiple subgroups, and individuals (perhaps in support of generic statements about the generic category) in multiple co-reference chains (Adolescent girls … , Lucy … , Mary … , Girls are …). The latter text provides a more nuanced (piecemeal) impression of individuals that may (or may not) be included in a social category. Note that a more nuanced text also facilitates, or reflects, alternative categorizations (e.g., adolescents and youngsters).

The linguistic content of references plays an important role in conveying and activating stereotype content. First, a single reference can include multiple categorizations for an individual. A reference like ‘the 30-year-old woman from Germany’ presents a nuanced intersectional impression of an individual, using age, gender, and origin, while a reference using a single category label (e.g., the German) reduces a person to just one dimension, which facilitates stereotyping. Second, the connotative meaning and valence of references are important. Labels with a derogatory (vs. positive or neutral) connotation convey negative affective evaluations and can reflect and maintain negative prejudice (Bilewicz et al., this volume; Carnaghi & Maass, 2007; Maass et al., 2014). In one co-refence chain, various labels can be used. After identifying a referent using a (more or less neutral) descriptive label (e.g., immigrants), additional labels (e.g., parasites and fortune-seekers) can qualify the category or specified members. Such negatively or positively connotated terms may induce or maintain stereotypic associations with the category immigrants.

The linguistic form of references also matters. References to a generic category may often use plural (pro)nouns (e.g., Athletes are … , They are). Particularly (plural) nouns, as opposed to references containing adjectives or descriptives, appear to reflect and induce higher levels of perceived category entitativity (Beukeboom et al., under review, Carnaghi et al., 2008; Rhodes et al., 2012). Yet, showing the versatility of language, singular references can also be used to refer to a generic category (e.g., A girl wears pink). Such expressions might reflect the category prototype (Zhang & Harwood, this volume). Subgroup references may often use constructions with adjective and noun (e.g., female athletes). Prepositional phrases within a reference (e.g., The man from the library; The people in Rotterdam) may specify a subset or individual. A prepositional phrase adds (situational) information about, e.g., place or time to references. It seems likely that such more nuanced references are used for new and/or low entitative categories (Beukeboom et al., under review).

With respect to the communication of stereotypes in descriptions of behavior and characteristics, research using the Linguistic Category Model (Semin & Fiedler, 1988) provided a major contribution. In the LCM approach, the speech act (the what) tends to be conflated with the linguistic form of the used predicates (the how): i.e., whether a description uses an action verb, a state verb or an adjective. This poses no problem in studies using experimenter-generated descriptions, in which sentences are carefully constructed to describe the same situation at different levels of abstraction, which are then rated or selected on their applicability in various situations (Maass, 1999). When applying the LCM on freely generated natural language texts, however, which multiple content analysis studies have done (Dragojevic et al., 2017; Maass, 1999; Menegatti et al., 2017; Rubini et al., 2009, 2014), it becomes clear that various creative linguistic tools can be used to achieve the same thing.

For instance, an action verb (e.g., run) can be used to describe a concrete observable behavior (e.g., He runs to his work), a mental state (e.g., In his mind, he runs through all the options), or a trait (e.g., He is a person who runs from responsibility). An action verb can also be part of a reference (e.g., [The guy who runs to work] was at my party). Moreover, prepositions can determine whether a behavior description refers to a specific situation in a specific point in time (e.g., The girl helped her mother yesterday) or generalize across an extended time period (e.g., The girl always helped her mother). When studying generalization in descriptions of behaviors and characteristics, it is therefore needed to look beyond the category of a predicate and annotate whether a behavior description in context refers to an observable action, a category of behaviors, a mental state, or a generic trait. It also important to link the behavior descriptions to the relevant target category reference (i.e., is a trait linked to a generic category or to a specified individual).

In our integrative approach, we look at both references and behavior descriptions. This is important, because particularly the combination of generic references (generalizing across individuals) with a characteristic that generalizes across situations (e.g., Boys play with trucks; Girls are sweet) are considered to play a crucial role in the transmission of category stereotypes (Cimpian & Markman, 2008; Gelman et al., 1998). In the literature, this has been studied as generic sentences, or generics, mostly within the developmental psychology field. Generic sentences explicitly convey that a given essential/enduring characteristic applies to an entire category. They are used frequently in child-directed speech and are important in children’s conceptual development (Gelman et al., 2010).

In natural language, generics can be expressed using various linguistic means. With bare plural nouns (e.g., Boys play with trucks), but also with definite or indefinite singulars (e.g., The adolescent is guided by peer pressure; A girl wears pink) and are accompanied by present-tense verbs (Gelman et al., 2004; Rhodes et al., 2012). Importantly, unlike utterances containing universal quantifiers such as all, every, or each, generic statements allow for exceptions, which makes them ideally suited to convey characteristics that are typical for a category, leaving aside few exceptions. For instance, a single counterexample would disprove the generalization ‘All boys play with trucks,’ but the generic statement ‘Boys play with trucks’ remains true even after an encounter with a single boy who does not. Arguably, this occurs because ‘All boys’ maintains the individuality of category members, while ‘Boys’ refers to the category as a single entity. Generics thus convey qualities that are stable (non-accidental), enduring, and persistent across time and situations (i.e., essential), and the generic reference also implies that the category is a coherent, stable entity (Beukeboom & Burgers, 2019; Gelman et al., 2004).

Various experimental recipient studies have shown effects of generics on stereotype communication. When characteristics of a (fictional) category were learned by means of generic sentences, respondents were likelier to later mention these characteristics as explanations for behavior of individual exemplars, to generalize them to novel exemplars, and to expect that category members are alike in other unmentioned characteristics as well (Gelman et al., 2010). Moreover, generics (compared to individual noun label and no-label) led to stronger perceived essentialism of characteristics; these were expected to be innate and inevitable, and stable across time (Gelman et al., 2010; Rhodes et al., 2012). Future research may explore whether different linguistic constructions in generic references relate to perceived entitativity. It seems plausible that definite singulars (e.g., The adolescent is guided by peer pressure) convey an even more entitative impression than plural generic references (e.g., Adolescents are …), because a singular label presents the category as one entity and strips away even further that it consists of multiple individuals.

Other linguistic elements than generalization in references and abstraction in behavior descriptions also play a role in communication of stereotypes, and these may complement the above. For instance, negations (e.g., the professor is not smart) likely play a role as these reflect (and maintain) the perception that a described behavior is stereotypically unexpected and a one-time event (Beukeboom et al., 2010, 2019). Aside from explicit negations (i.e., using not), natural language descriptions may contain alternative ways to express negation (Horn, 1989). Moreover, there may be other explicit or implicit expressions that can communicate whether described behavior of (members of) the target category is stereotypically expected (e.g., as usual) or seen as an unexpected one-time event (e.g., unexpectedly).

## When do we generalize in communication and share stereotypes?

What are the factors that make people generalize across individuals, situations, and time in their communication rather than specify and use more nuanced descriptions?

Following the SCSC framework (Beukeboom & Burgers, 2019), generalization is facilitated with higher perceived category entitativity, and higher perceived essentialism of behaviors and characteristics. This means that stereotypic information is more likely communicated using generalized language, which in turn communicates it to message recipients. This occurs for various reasons.

First, cognitive processes of the sender in processing communicated social information play a role. Language use is often a reflection of the sender’s ongoing cognitive processes and representation of social knowledge.^3^^3^Language use is not merely a reflection of cognitive processes. For instance, in intergroup situations, motivational factors and communication goals related to a speaker’s social identity can induce biased language use in comments about behaviors by in- and out-group members, and this can override effects of cognitive expectancies (Douglas & Sutton, 2003; Maass et al., 1995, 1996). When people process social information, they can do this in a low-effort, superficial manner; information is then represented and processed at a generic level (Fiske & Neuberg, 1990). With enough time, cognitive resources, and motivation, however, people can tune to processing information more deeply. They will then seek more specificity in fine-grained, subordinate categorization with higher granularity (e.g., subtyping), or ultimately a piecemeal processing of individual attributes (Fiske & Neuberg, 1990; Hamilton & Sherman, 1996). This reflects a narrowing process toward lower, more specific levels of information (cf. Beike & Sherman, 1994; Bolognesi et al., 2020), which contrasts the opposite tendency toward greater generalization. When people communicate about social information, their language will reflect such processes and subsequently induce changes in the language recipient.

In processing social information, there is a default level of generalization. When people try to make sense of social information, people seek the right level of specificity in the (social) categories they apply. Basic-level categories are seen as the sweet spot between too vague/generic and too specific, and the most cognitively efficient and ‘natural’ level for categorization (Rosch et al., 1976). By using category references and labels, this is expressed in language (Brown, 1958). The basic level has been termed the ‘level of usual utility,’ which is the default or most commonly used level that people naturally use when naming or referring to things in everyday communication to achieve social coordination (Enfield, 2022).

The default level may vary by social context. This is, firstly, visible in the lexicon in different communities. Variations in the granularity of common categories show that people develop more specific categories at a lower level of generalization when needed for social coordination (Enfield, 2022). For instance, people living in nature have more fine-grained tree terms than urban dwellers, and surfers develop nuanced labels for waves (Hunt & Banaji, 1988). The same can be expected for social categories. Experts working in the health industry likely categorize professions and roles using multiple specific categories (e.g., anesthesiologist, radiologist, and paramedic), while outsiders settle with a more limited number of categories at a higher level of generalization (e.g., doctors and nurses). This aligns with the outgroup homogeneity effect (Park & Rothbart, 1982) showing that people see (and likely communicate) less diversity and individual variation among outgroup members than among ingroup members.

Second, the level of generalization can be adapted in ongoing online processes depending on the context. In processing observed behavior and characteristics of categorized target persons, people will seek the most fitting category and associated stereotype to maximize the accuracy of predictive inference (i.e., the most information gain; Anderson, 1991; Hamilton & Sherman, 1996). When inconsistencies are perceived between a target person’s features and behavior and an activated category representation (i.e., fit is low; e.g., a person does not show stereotypical characteristics), this will prompt a perceiver to process more deeply. This search for a more fitting categorization may result in re-categorization or subtyping, or ultimately (with enough time, resources, and motivation) a narrowing to a piecemeal processing of individual attributes (Fiske & Neuberg, 1990; Hamilton & Sherman, 1996). Social information that is out of the ordinary can thus be expected to be discussed at lower levels of generalization than information that is congruent with a generic stereotype (Beukeboom & Burgers, 2019).

In line with this notion, research on the Linguistic Intergroup Bias (LIB; Maass, 1999; Maass et al., 1995) and Linguistic Expectancy Bias (LEB; Rubini & Semin, 1994; Wigboldus et al., 2000) has demonstrated that people tend to use more concrete language to describe stereotype-inconsistent behavior (e.g., the man is crying), while stereotype-consistent behavior is described abstractly (e.g., the woman is emotional). Moreover, stereotype inconsistent (vs. consistent) information is elaborated more, which results in longer sentences (Eskreis-Winkler & Fishbach, 2022) and added explanations (Sekaquaptewa et al., 2003).

Research on interpersonal communication shows the interactivity of these processes. For any generalization in language, agreement on its meaning is necessary. To achieve mutual understanding, we need to have a socially verified common ground about generic/abstract terms, otherwise they are useless for communication. New categorizations that are not yet part of a shared reality thus need to be socially verified (Kashima et al., 2007; Klein et al., 2008). When new categories are formed, we likely start at specific level, before we can agree on a generic label (a.k.a. conceptual pacts; Brennan & Clark, 1996). For instance, an attempt to categorize observations may develop from descriptive references including prepositional phrases (e.g., ‘those people who are protesting in Paris and wearing yellow vests’) to the use of conceptual pacts expressed in a generic noun (e.g., the yellow-vests). In this grounding process, we collaboratively form a common ground for communication at a higher level of generalization.

When we have a shared understanding of common labels, we still need to tune our level of generalization to what is needed in the interaction that we are in. To maintain mutual understanding, our linguistic utterances need to hit a happy medium between being not too vague, and no more specific than needed (Grice, 1975). Conversation partners collaborate in achieving the right level of specificity in a grounding process (Clark, 1996; Clark & Brennan, 1991). We can communicate at a relatively high level of generalization (e.g., basic-level categories) when we understand and agree with each other. This would also be the preferred and default level as it is most efficient (i.e., principle of least collaborative effort). When needed, however, for instance, when a conversation partner signals misunderstanding or disagreement on a categorization (e.g., frowns, critical questions), a speaker will move to a lower level of generalization (Krauss & Weinheimer, 1966). We then need to go back to more nuanced information and provide concrete explanations or evidence to justify a generic statement.

However, in situations of like-minded people (i.e., ingroup members who agree in their impression of a certain outgroup, and no critical questions or disagreement are uttered), communication can occur at a higher level of generalization. This is the type of situation where people can quite directly share generic stereotypes in language (e.g., They are …), occurring for instance in a bar among friends, in locker-room talk, or in like-minded social media groups (i.e., echo chambers).

## Discussion

In this article, I aimed to integrate existing knowledge about stereotype communication by focusing on generalization in natural language use. We generalize by placing information into a limited number of higher level categories, in order to understand our complex and diverse social environment. This type of interpretation into categories is a strong human tendency which we do collaboratively when we discuss other people and their behavior. Social-category stereotypes are communicated through generalized language (e.g., girls are sweet). It reflects and maintains the impression that the information applies across individuals and across situations and time. When we generalize, we also over-simplify information; it lacks nuance as it moves away from specific individuals and concrete behavioral situations.

Generalization is not necessarily harmful. Categorizing in generic terms reduces complexities and this is often functional, as it gives a sense of reassurance and ability to predict (Mackie et al., 1996). It helps us to make sense of an otherwise complex social world by conveniently organizing it in a manageable number of boxes. It can, however, be harmful when we forget that it lacks nuance, and when we confidently base our judgments of others and their behavior upon this limited representation of reality. Discussing social information at a generic level strips away specific distinguishing information about individuals and situations. When we label a group of people in one category, we treat a bunch of unalike individuals as being the same and ignore the differences among them (Enfield, 2022). A single generic label (e.g., immigrants) reduces the group to just this one categorizing feature. And when we talk about behavior at a generic level (e.g., aggressive, sweet), we imply that it is enduring and expected across situations and time, while there may be little or no basis for this in reality.

When we rely on such superficial generic information to judge and treat individuals this results in prejudice and discrimination. Prejudice, hate speech (Bilewitz et al., this volume), discrimination, and even violence against minority groups are most likely to emerge when perceptions of strong entitativity are formed, with negative essentialized stereotypic impressions (Beukeboom & Burgers, 2019). Moreover, labeling generic groups (e.g., boys vs. girls) can enhance intergroup competition and conflict as it reduces complexity to black and white and exaggerates differences between categories. An integrated and in-depth understanding of generalization in natural language can help us uncover such processes in everyday natural language.

### Future research directions

A first avenue for future research relates to the development and use of computational tools for automated detection of stereotyping in natural language texts. With the rapid development of natural language processing techniques (Boyd & Markowitz, 2025), such tools could offer unprecedented opportunities for further (applied) research and content monitoring in a variety of real-life contexts (see also, Giles et al., this volume). At the same time, I showed why this is challenging, given the creative and complex constructions people use, and the fact that their meaning depends on the broader context. Consequently, automated methods using dictionary approaches that merely rely on word categories and syntax (counting descriptive action verbs etc., e.g., Collins & Boyd, 2025) are limited as the broader context is not considered. That is, words are embedded in sentences, sentences in texts, texts in genres (e.g., interactive conversations, social media post, news article, organizational document). In addition, intrapersonal factors (e.g., mood, motivations, identity), interpersonal and intergroup relationships (e.g., is it communication *between* social groups or *about* a third category, see Zhang & Harwood, this volume), and the societal context (e.g., social history; are participants part of majority, minority) may play a role. For a comprehensive understanding, NLP tools are best developed with a transdisciplinary approach that combines strong theoretical grounding with attention to multiple influencing factors (Boyd & Markowitz, 2025).

A somewhat related question is to what extent the described processes are present in different languages, and, if they are shared, how they are realized; note that I mainly focused on structural aspects of language, often related to underlying functions of language use. Most languages, if not all, have means to generalize and/or provide concrete nuance, to express negation, or to indicate whether things are expected or not. The ways in which this is linguistically achieved, however, may vary per language. This means that guidelines for manual annotation and computational tools will have to be (at least partly) developed and validated separately for different languages. For that, a thorough understanding of the various aspects of language through which stereotypes are communicated is needed. Future research should further explore this and study which computational techniques can be implemented effectively.

A second avenue of research relates to interactive processes that are at the heart of intergroup communication field (Giles, prolog this volume). Language plays a central role in interactive intergroup communication, by marking social categories and shaping individuals’ identity and sense of belonging (Hogg & Reid, 2006; Tajfel & Turner, 1986; Turner et al., 1987). First, in describing ourselves, we can affirm and enhance our social identity and in-groups, and simultaneously distance ourselves from outgroups (Dragojevic et al., 2015; Maass et al., 1996). Second, language used to refer to and address others in our presence can immediately activate certain social identities. A quick remark can prime one social identity over another (e.g., gender, ethnicity, and organizational department) and establish a comparative intergroup context. For instance, referring pronouns (e.g., you vs. we, us vs. them) play a big part in communicating which categories apparently matter and who belongs to in- and outgroups. Also, addressing people with binary gender labels (e.g., ladies and gentlemen) activates an often-irrelevant gender categorization and excludes nonbinary persons (see also Palomares, this volume; Zhang & Harwood, this volume). Choosing other, non-gendered labels (e.g., dear customers) can prevent that. Third, even without explicitly labeling/categorizing people, language can subtly reflect and activate stereotypic expectancies, for instance, in the questions asked (Beukeboom et al., 2025). Being stereotyped – albeit by means of subtle linguistic cues – can in turn induce people to confirm expectancies as a self-fulfilling prophecy (Hummert et al., 2004), can induce impaired performance as a result of stereotype threat (Steele, 1997; von Hippel et al., 2024), or improve performance as a result of stereotype lift (Walton & Cohen, 2003).

Understanding the role of various aspects of language can help to raise awareness of such processes, and an ability to detect them in discourse. This, in turn, can help to change or prevent social exclusion and the formation and maintenance of undesirable category stereotypes. In various work contexts (Kurylo & Hu, 2024) language policies or instructions could be effective in preventing harmful effects by facilitating the use of proper language. This could relate to the language used to refer to and describe groups in (news) media or official forms or texts, or to address citizens, customers, or employees. Such policies can promote the use of inclusive language over terms that exclude, unnecessarily categorize, or reinforce intergroup distinctions, while discouraging superficial generalizations that sustain stereotypes.

Future research can also help to determine appropriate interventions to turn overly generic language use (e.g., stereotypic statements) to more nuance. The research on conversational processes that I discussed (e.g., Clark, 1996) shows that too much generalization can be countered by a simple frown, a silence, or a question for substantiation (e.g., ‘what is a that based upon?,’ ‘How do you know?’). This forces a speaker toward more nuance and concrete detail. With such responses, we can make each other aware when communication is superficially generic and lacks concrete substantiation.

## Conclusion

To conclude, understanding how stereotypes are communicated in natural language requires an integrative approach, which I pursued by focusing on generalization in both references and behavior descriptions. I hope this contributes to greater awareness of unnecessary generalization, and supports efforts to detect, prevent, and challenge unwanted stereotyping in everyday communication.
